# Cosmogony

**Published:** 1884-04

**Authors:** J. B. Chandler


### COSMOGONY.

J. B. CHANDLER.

"In the beginning God created the Heaven and the Earth. And the Earth was without form, and void."

These words are specially familiar to me; as a child they came with scriptural home teachings; in later years I have used them frequently on most solemn occasions; yet, their significance as to *how* the world was made was lost in the great fact announced, by Whom it was made.

After reading with some care a most interesting book* by Prof. Alexander Winchell, LL.D., of the University of Michigan, I am led to note that the writer of Genesis, whether inspired or not, uses language which might seem to imply the previous existence of the Earth in a state which only required the movement of the Spirit for it to assume *form* and order and take its place amongst the heavenly bodies.

Its freshness to my brain is perhaps the only novelty about this thought. Because this book furnishes food for thought, (whether such thinking may or may not lead to the adoption of the theory of Nebulæ evolution) I have deemed it not unworthy the notice of the inquiring readers of the BISTOURY.

No fear need be entertained that the work of which I write, or the theory which it explains, have any antagonism to the sound principles of religion, or assume the denial of a Great First Cause, "Jehovah, Jove, or Lord."

I quote from the Book :—

"These thoughts summon into our immediate presence the measureless past and the measureless future of material history. They seem almost to open vistas through infinity and to endow the human intellect with an existence and a vision exempt from the limitations of time and space and finite consideration, and lift it up toward a sublime apprehension of the Supreme Intelligence whose dwelling place is Eternity."

The theory of a vortical conception was entertained 500 years B. C. Leucippus (430 B. C.), maintained "that space was eternally filled with atoms actuated by an eternal motion," and that certain laws of *weight* (not then understood), gradually produced a rotary motion which in time resulted in the formation of worlds.

Farther down, into the sixteenth century, we find the speculations of Kepler. In the next century was promulgated the Vortical Theory of Descartes. Leibnitz gave us the theory of a molten world with a wonderful and ingenious deduction of the present Earth therefrom.

Swedenborg published in 1733 a work on the "Principles of Things," in which a vortical theory was set forth. Then we have the Theory of Kant, and the Researches of Sir William Herschel.

La Place's "System of the World" was written in popular style, presenting the general results of astronomical research; in which occurs his Hypothesis of the Genesis of Solar System from Nebulæ.

This hypothesis is nearest to the theory of which Prof. Winchell treats and which may be summed up in plain language, thus:

The Universe, as we now contemplate it, has resulted gradually from the combination of its elements which existed in a chaotic state in space, and this, under the immutable laws of God, the action of some of which, such as gravitation, centripetal and centrifugal forces, are known to us, and offer means through which the result might have been produced.

The chaotic state of elementary matter is represented by the immense masses of nebulæ visible now from the earth, but not resolvable by any telescope yet manufactured.

The "Milky Way" has been satisfactorily resolved and ascertained to be stellar; but there exist nebulæ within reach of the powers used which although proven by the Spectroscope to be composed of elements which go to make up worlds, are yet in process of formation into systems. In other words, creation is not yet

*World-life or Comparative Geology.

S. C. Griggs & Co., Chicago.

complete, but constantly progressing, and, what to those who have given these things little thought, may appear most strange, growth and decay are going on amidst the spheres. New worlds forming, old ones decaying. Our world has its period, and even now mathematical minds are calculating its duration in years. The theory that nebulæ are masses of cosmic dust has been doubted because, since it was first entertained, the improvement in telescopes has resulted in the resolution of some into groups of stars so distant and so numerous that to low powers they appeared only as brilliant clouds. Notwithstanding this, careful observations have produced sufficient reasons for the placing of the nebulæ hypothesis upon the basis of a scientific theory.

These reasons are plainly and scientifically given by Prof. Winchell in his work, and the book is replete with diagrams and illustrations; to aid the reader.

Whether God made the world in three days, or whether it was formed in conformity with natural mechanical laws emanating from the "Supreme Intelligence" in the time required by the action of those laws, seems to be a matter not bearing much upon the present or future of the people of the nineteenth century ; but the fact that the researches of scientific men have reached a point which enables them to present such a theory carried by proof far beyond mere speculation, should be generally known.

The book under consideration is within the reach of those who, seeking to know more of the subject than this short sketch can give, may desire to follow the author through the interesting course of study and proof which he brings forward in defense of the theory. It deals with the subject not only scientifically but also plainly enough to adapt the reasoning to the comprehension of the general reader.

There be those of ability who will not agree with Prof. Winchell, and who perhaps have strong points to advance against his arguments, which we of the laity in science know not. Those who are fortunate enough to acquire the arguments on both sides will be no worse for the mental effort.

The blacksmith develops muscle by its use, the reader will not find his brain weakened by such a study as Cosmogony.

Such speculations, even if they amount to theories, are apt to shock those whose minds have been narrowed to a simple acceptance of teachings which grew out of the want, or the suppression, of knowledge because of the fear of entrusting to the general public any discovery which might disturb settled convictions.

The days of such a policy are passing away; education is doing its work and the people are fast acquiring the ability to shake off the rule which would keep them in the control of ignorance.

The Nebulæ Theory may or may not be correct, but the study of it can hurt no one. The wonderful works of the Almighty, as far as we are permitted to know them, cannot fail to impress the student, the farther he advances, with wonder, awe and reverence for that Great Cause :

> "Who sees with equal eye, as God of all,
> A hero perish, or a sparrow fall,
> Atoms or systems into ruin hurled,
> And now a bubble burst, and now a world."

